# Latent profiles and associated factors of readiness for hospital discharge among patients undergoing orthognathic surgery: a cross-sectional study

**DOI:** 10.3389/fpubh.2026.1925469

**Published:** 2026-09-24

**Authors:** Yuan Liu, Yuncai Chen, Ao Huang, Xingyue Chen, Yonglu Huang, Bei Wang, Li Li

**Affiliations:** 1 Department of Oral Nursing Teaching and Research, Nanjing Stomatological Hospital, Affiliated Hospital of Medical School, Nanjing University, Nanjing, China; 2 Institute of Stomatology, Nanjing University, Nanjing, China; 3 School of Nursing, University of South China, Hengyang, China; 4 Medical School of Nanjing University, Nanjing, China; 5 Nanjing Stomatological Hospital, Affiliated Hospital of Medical School, Nanjing University, Nanjing, China

**Keywords:** latent profile analysis, orthognathic, orthognathic surgery, patient, readiness for hospital discharge

## Abstract

**Objective:**

This study aims to identify the latent profiles of readiness for hospital discharge among patients with orthognathic conditions and to explore associated factors, thereby providing a basis for developing targeted discharge-preparation nursing interventions in clinical practice.

**Methods:**

Using convenience sampling, this study recruited patients undergoing orthognathic surgery who were admitted to a tertiary stomatological hospital in Nanjing, Jiangsu Province, China, between March and September 2025. Data were collected using a General Information Questionnaire, the Chinese version of the Orthognathic Quality of Life Questionnaire, the Quality of Discharge Teaching Scale, and the Readiness for Hospital Discharge Scale. Latent profile analysis was conducted using the three RHDS dimension mean scores as nonredundant indicators. Univariate analyses, multiplicity-adjusted post-hoc comparisons, and multivariable multinomial logistic regression with sensitivity analyses were used to examine factors associated with assigned profile membership.

**Results:**

The median RHDS mean score was 7.50 (5.98, 8.25). Latent profile analysis identified three distinct profiles after reordering the Mplus classes for interpretability: low readiness-comprehensively weak (*n* = 103, 25.0%), moderate readiness-support advantage (*n* = 141, 34.2%), and high readiness-adaptation leading (*n* = 168, 40.8%). Quality of discharge teaching, quality of life, living status, household income, educational attainment, and type of surgery were associated with profile membership in the multivariable analysis.

**Conclusion:**

Readiness for hospital discharge among orthognathic patients was moderate and heterogeneous. These findings suggest that profile-informed discharge assessment may help identify patients who need additional support; however, longitudinal and interventional studies are required to determine whether profile-based discharge-preparation strategies improve post-discharge outcomes.

## 1 Introduction

Dentofacial deformities are developmental abnormalities of the teeth, jaws, and facial structures that result in significant deviations from normal maxillomandibular relationships, occlusion, and facial proportions (1). These deformities not only affect patients’ mastication, speech, and oral function (2), but also impair facial esthetics, self-image, social interaction, and oral health-related quality of life (3, 4). Previous studies have shown that patients with dentofacial deformities frequently experience psychosocial difficulties, such as reduced self-esteem, social avoidance, and depression (5–7).

For skeletally mature adult patients with severe dentofacial disharmony, orthognathic surgery is required. Orthognathic surgery is one of the most commonly performed and standardized procedures in maxillofacial surgery (8). Since the beginning of the 21st century, research attention has gradually shifted from traditional clinical outcomes, such as esthetics, function, and complications, toward OHRQoL in order to better incorporate patients’ perspectives (9). Improving quality of life has become a key objective of orthognathic treatment (10).

In recent years, enhanced recovery after surgery (ERAS) protocols have been increasingly implemented in oral and maxillofacial surgery, including the perioperative management of patients undergoing orthognathic surgery. A retrospective comparative study by Wahlstrom et al., (11) suggested that ERAS protocols can reduce the maximum pain scores of orthognathic patients. The evidence synthesis by Joachim and Miloro (12) also indicated that ERAS protocols for orthognathic surgery have practical value in multimodal analgesia, prevention of postoperative nausea and vomiting, early nutrition, and standardized discharge. However, the reduced length of hospital stay associated with ERAS may result in patients returning home before achieving complete recovery. Moreover, in the context of China’s new healthcare reform, patients’ length of hospital stay has decreased from the traditional approximately two weeks to one week or even shorter. At discharge, patients may still have insufficient ability to perform activities of daily living, and home-based rehabilitation after discharge may be inadequately implemented (13), thereby increasing the risk of post-discharge complications. Therefore, assessing whether orthognathic patients possess adequate readiness for hospital discharge, including sufficient physical status, self-care capability, and psychological adaptability, has become increasingly important for ensuring safe discharge and continuity of rehabilitation.

Readiness for hospital discharge refers to patients’ overall perception of whether they possess adequate physical condition, self-management capability, home-care resources, and rehabilitation support before leaving the hospital. (14). For orthognathic patients, discharge readiness is closely related to whether they can successfully complete the transition from ‘hospital-based care’ to ‘self-management’.

At present, studies on orthognathic patients have mainly focused on surgical techniques, postoperative complications, and quality of life. However, research on readiness for hospital discharge among orthognathic patients remains relatively insufficient. Existing studies often treat readiness for hospital discharge as a continuous variable and describe its overall level, while paying limited attention to whether heterogeneous subgroups with different care needs exist within the patient population. In fact, orthognathic patients differ considerably in terms of surgical complexity, perceived quality of life, financial burden, and the extent to which they receive discharge teaching. Evaluating readiness for hospital discharge solely on the basis of average levels may may obscure clinically meaningful heterogeneity and limit the development of targeted nursing interventions.

Therefore, the present study applied latent profile analysis (LPA) to identify distinct readiness-for-discharge profiles among orthognathic patients and to examine factors associated with profile membership. LPA is a person-centered statistical method that can identify subgroups with similar characteristics based on patients’ response patterns across multiple dimensions, helping shift the focus from mean group differences to individualized risk identification. By identifying latent subgroups with distinct characteristics, this study provides evidence for the development of precision discharge education, individualized rehabilitation support, and profile-based transitional care strategies under the ERAS framework, ultimately promoting safer transitions to home-based recovery and improving perioperative nursing outcomes for patients undergoing orthognathic surgery.

## 2 Subjects and methods

### 2.1 Survey participants

Convenience sampling was used to recruit orthognathic patients admitted to a tertiary stomatological hospital in Nanjing, from March to September 2025 as the study participants. The inclusion criteria were: (1) age < 50 years; (2) scheduled to undergo or having undergone combined orthodontic-orthognathic treatment; (3) educational attainment of at least junior high school level; (4) absence of cognitive impairment and ability to communicate effectively; and (5) provision of informed consent as approved by the ethics committee. The exclusion criteria were: (1) severe dysfunction of major organs (e.g., heart, liver, or kidney); (2) diagnosed cognitive impairment or psychiatric disorders; and (3) incomplete questionnaire data.

Before recruitment, the target sample size was determined pragmatically because the number of latent profiles was unknown *a priori* and no universally accepted closed-form sample-size formula exists for exploratory latent profile analysis. Methodological studies indicate that sample-size requirements in mixture models depend on several unknown population parameters, including class separation, class proportions, number and quality of indicators, and model complexity. Therefore, a conventional power calculation for a prespecified number of profiles was not applicable. Following applied LPA practice and methodological recommendations that exploratory latent profile models generally require large samples, with samples of at least 300 considered desirable and samples around 400–500 commonly used in applied LPA studies (15), we set a minimum target of 400 valid cases. This target also exceeded the conventional requirement for cross-sectional multivariable studies, in which the sample size is often estimated as 10–15 times the number of observed study variables, with an additional allowance for invalid or incomplete questionnaires (16). Ultimately, 412 participants with complete data were included.

#### 2.1.1 Ethical considerations

This study was conducted in accordance with the Declaration of Helsinki and was approved by the Ethics Committee of the Stomatological Hospital of Medical School of Nanjing University (approval number: NJSH-2025NL-012). The requirement for written signed informed consent was waived by the ethics committee because this was a minimal-risk questionnaire-based study. Before completing the electronic questionnaire, all participants and, where applicable, their parents or legal guardians were informed of the study purpose, voluntary nature of participation, confidentiality, and right to withdraw. Electronic informed consent was obtained from adult participants. For participants younger than 18 years, both the participant and their parent or legal guardian were informed of the study, and consent was obtained from the parent or legal guardian together with assent from the participant. The electronic informed consent form for minors was completed and confirmed by the parent or legal guardian.

### 2.2 Measures

#### 2.2.1 General information questionnaire

The researchers created a demographic information questionnaire, including sex, age, admission weight, discharge weight, height, occupation, place of residence, marital status, living status, educational attainment, and annual household income level. Additionally, information related to orthognathic treatment was collected, including the length of hospital stay following orthognathic surgery, the type of orthognathic surgery performed, and the method of medical expense reimbursement.

#### 2.2.2 Orthognathic Quality of Life Questionnaire

The Orthognathic Quality of Life Questionnaire (OQLQ) was developed in 2000 to assess the quality of life of patients with dentomaxillofacial deformities and the impact of surgical treatment (17). Following translation and validation, the Chinese version of the OQLQ (OQLQ-C; (18)) consists of 4 dimensions: oral function (5 items), facial esthetics (5 items), awareness of facial deformity (4 items), social aspects of deformity (8 items), for a total of 22 items. Each item is rated on a 5-point Likert scale ranging from 0 (‘does not bother you’) to 4 (‘bothers you a lot’); the total scale score ranges from 0–88 points. A higher total score indicates poorer quality of life in orthognathic patients. The Cronbach’s α coefficient of the Chinese OQLQ was 0.932 (18). In this study, the Cronbach’s α coefficient of this scale was 0.894.

#### 2.2.3 Quality of Discharge Teaching Scale

The Quality of Discharge Teaching Scale (QDTS) was developed to assess patients’ perceptions of the quality of discharge teaching received prior to hospital discharge (14). The Chinese version of the QDTS scale, which has been translated and validated (19), includes 3 dimensions: pre-discharge needs (6 items), actual outcomes (6 items), and teaching skills and effectiveness (12 items), for a total of 24 items. The 12 items in the first two dimensions form six pairs of parallel items. The scale measures the quality of discharge teaching by calculating the total score across two dimensions: the actual outcomes and the teaching skills and effectiveness. Each item is scored from 0 to 10, and the total score is the sum of the scores for these two dimensions. The possible total score ranges from 0 to 180, with higher scores indicating better perceived quality of discharge teaching. The Cronbach’s α coefficient of the Chinese QDTS was 0.924 (19). In this study, the Cronbach’s α coefficient of this scale was 0.923.

#### 2.2.4 Readiness for Hospital Discharge Scale

The Readiness for Hospital Discharge Scale (RHDS) was developed in 2006 to assess the readiness of hospitalized patients for discharge (14). Following translation and validation, the Chinese version of the RHDS (20) consists of 12 items across 3 dimensions: personal status (3 items), adaptive ability (5 items), expected support (4 items). Each item is scored from 0 to 10, with a total scale score of 0–120. The three RHDS indicators used in the LPA were calculated as dimension mean scores: personal status = Σ(items 1–3) / 3, adaptive ability = Σ(items 4–8) / 5, and expected social support = Σ(items 9–12) / 4. Each dimension mean score ranges from 0 to 10, with higher scores indicating greater perceived readiness for discharge. The Cronbach’s α coefficient of the Chinese RHDS was 0.89 (21). In this study, the Cronbach’s α coefficient of this scale was 0.87.

### 2.3 Data collection and quality control methods

Participants were recruited in strict accordance with the predefined inclusion and exclusion criteria. Data were collected using an electronic questionnaire administered through Questionnaire Star by uniformly trained investigators. Questionnaire items were configured as required fields in the electronic platform; nevertheless, five records were excluded because the submitted records contained incomplete analyzable data during the final verification step. Unique IP addresses were restricted to a single submission, and response completion times were recorded. Questionnaires completed in less than 2 min were regarded as invalid. Electronic questionnaires were distributed to eligible patients on the day of discharge or one day before discharge after routine discharge teaching had been completed. Participants completed the questionnaires anonymously and independently; for patients who had difficulty completing the questionnaire independently, investigators recorded responses according to the participant’s oral answers.

### 2.4 Statistical methods

Excel 2016 was used for data organization and verification. Descriptive statistics, bivariate group comparisons, and multinomial logistic regression were conducted using SPSS 27.0 and independently checked against the source dataset. Latent profile analysis was performed in Mplus 8.3. The three RHDS dimension mean scores (personal status, adaptive ability, and expected social support) served as the LPA indicators; the overall RHDS score was used only for descriptive reporting and was not entered as a redundant indicator. Models with 2 to 5 latent profiles were estimated using robust maximum likelihood (MLR) with 1,000 initial-stage and 250 final-stage random starts to mitigate local maxima, assuming equal variances and zero covariances (local independence) across classes. Missing data (−999) were handled via FIML; convergence was set to default (relative LL change < 1e-6) with STITERATIONS = 20. *p* < 0.05 (two-tailed) indicates statistical significance (22).

Model selection followed established criteria (23): (1) Information evaluation indices included the Akaike information criterion (AIC), Bayesian information criterion (BIC), and adjusted Bayesian information criterion (aBIC), with smaller values indicating better model fit. (2) Classification evaluation indices used entropy to assess classification accuracy. The closer the entropy value is to 1, the more precise the classification. When the entropy is ≥0.8, it indicates that the classification accuracy exceeds 90%. (3) Use the L-Mendell–Rubin adjusted likelihood ratio test (LMRT) and the Bootstrapped likelihood ratio test (BLRT) to compare the differences in goodness of fit between the k-category model and the (k-1)-category model. If *p* < 0.05, this indicates that the k-category model is superior to the (k-1)-category model.

Model selection considered AIC, BIC, sample-size-adjusted BIC, LMRT, BLRT, entropy, average posterior probabilities, class size, parsimony, and clinical interpretability. Entropy was interpreted as an index of class separation and classification certainty, not as a percentage of correctly classified participants. For variables with significant omnibus tests across profiles, Dunn-Holm post-hoc tests were used after Kruskal-Wallis tests, and adjusted pairwise comparisons were used for categorical variables. Local independence was evaluated by inspecting the within-class residual/correlation output, and no large residual association suggesting serious local dependence was identified. Entropy was reported as an index of profile separation and classification certainty rather than as a direct classification-accuracy percentage.

Multivariable model variables were selected based on clinical relevance, prior evidence, and descriptive results rather than univariate *p* values alone. QDTS and OQLQ scores were rescaled per 10-point increase, and household income, educational attainment, and surgery type were entered as indicator variables with explicit reference categories. Classification uncertainty was examined through sensitivity analyses restricted to participants with maximum posterior probability ≥ 0.80 and through posterior-probability-weighted multinomial logistic regression.

Quantitative variables were summarized as mean ± standard deviation or median (P25, P75), as appropriate. Between-profile comparisons used analysis of variance or Kruskal-Wallis tests for quantitative variables and chi-square, Fisher exact, or Monte Carlo exact tests for categorical variables, depending on expected cell counts. Significant omnibus tests were followed by multiplicity-adjusted pairwise comparisons. Multinomial logistic regression was used to examine factors associated with assigned profile membership. Two-sided *p* < 0.05 was considered statistically significant.

## 3 Results

### 3.1 General information of respondents

A total of 428 participants were recruited. After data screening, 16 participants were excluded, leaving 412 participants for the final analysis (effective response rate: 96.3%). Reasons for exclusion included incomplete questionnaire data (*n* = 5), invalid responses (*n* = 8), and failure to meet the inclusion criteria (*n* = 3). Most participants were female (324, 78.6%) and aged 18–25 years (286, 69.4%). The majority were unmarried (351, 85.2%), lived with others (298, 72.3%), and resided in urban areas (298, 72.3%). Most participants had received college education or above (335, 81.3%), and self-payment was the predominant method of medical expense coverage (367, 89.1%). The median RHDS mean score was 7.50 (5.98, 8.25), with a personal status score of 6.33 (5.00, 8.33), an adaptive ability score of 7.40 (5.60, 8.80), and an expected social support score of 8.00 (6.00, 9.25). Other demographic and clinical characteristics are presented in Table 1.

**Table 1:** Baseline characteristics of the study population (*n* = 412).

Variable	No. (%) or median (IQR)
Gender	
Female	324 (78.6)
Male	88 (21.4)
Age (years)	
<18	4 (1.0)
18–25	286 (69.4)
26–30	90 (21.8)
31–40	32 (7.8)
Spouse status	
No spouse	351 (85.2)
Have a spouse	61 (14.8)
Height (cm)	
150–160	62 (15.0)
160–170	226 (54.9)
170–180	95 (23.1)
≥ 180	29 (7.0)
Home residence	
Urban area	298 (72.3)
Town or rural area	114 (27.7)
Residence status	
Living alone	114 (27.7)
Not living alone	298 (72.3)
Educational attainment	
High school and below	77 (18.7)
Undergraduate	241 (58.5)
Postgraduate and above	94 (22.8)
Occupation	
Employees	80 (19.4)
Self-employed	78 (18.9)
Students	157 (38.1)
Civil servant	10 (2.4)
Teacher	21 (5.1)
Others	66 (16.0)
Monthly household income (CNY)	
1,000–3,000	9 (2.2)
3,000–5,000	76 (18.4)
5,000–10,000	150 (36.4)
>10,000	177 (43.0)
Health insurance type	
All out of pocket	367 (89.1)
Urban and rural residents’ medical insurance	32 (7.8)
Others	13 (3.2)
Length of hospital stay (day)	
0–5	77 (18.7)
6–10	317 (76.9)
11–15	15 (3.6)
Weight on admission (kg)	
40–55	156 (37.9)
55–70	178 (43.2)
70–85	58 (14.1)
85–100	20 (4.9)
Weight at discharge (kg)	
<40	10 (2.4)
40–55	192 (46.6)
55–70	154 (37.4)
70–85	48 (11.7)
85–100	8 (1.9)
Type of surgery	
Monomaxillary surgery	78 (18.9)
Monomaxillary surgery & genioplasty	2 (0.5)
Bimaxillary surgery	237 (57.5)
Bimaxillary surgery & genioplasty	94 (22.8)
Other surgery	1 (0.2)

### 3.2 LPA and nomenclature of readiness for hospital discharge in orthognathic surgery patients

Five latent profile models were evaluated, with results shown in Table 2. AIC, BIC, and aBIC decreased as the number of classes increased. The three-profile solution showed good separation (entropy = 0.906), high average posterior probabilities for most likely class membership (0.954, 0.973, and 0.966 for the three Mplus classes), and clinically interpretable patterns. The four-profile model showed significant LMRT and BLRT results, and its best log-likelihood was successfully replicated using 2,000 initial-stage and 500 final-stage random starts. However, the additional fourth profile contained only 15 assigned participants (3.6%; estimated *n* = 18.37, 4.5%). Although this small profile presented a pattern of relatively low personal status and expected social support but moderate adaptive ability, it did not represent a clinically or theoretically distinct readiness state beyond the three-profile solution. Therefore, despite a high classification quality (entropy = 0.907) and stable convergence, the three-profile solution was retained as the final model to ensure statistical parsimony, adequate profile sizes, and clear clinical interpretability.

**Table 2:** Model fitting results of latent profile analysis of discharge readiness among orthognathic patients (*n* = 412).

Class	Loglikelihood (LL)	AIC	BIC	aBIC	Entropy	LMRT (*P*)	BLRT (*P*)	Category
1	−2654.649	5321.298	5345.424	5326.385	-	-	-	-
2	−2497.215	5014.429	5054.640	5022.907	0.792	0.093	<0.001	40.8/59.2
3	−2365.970	4759.941	4816.235	4771.810	0.906	<0.001	<0.001	25.0/34.2/40.8
4	−2346.300	4728.599	4800.978	4743.860	0.907	0.019	<0.001	3.6/21.8/34.0/40.5
5	−2326.586	4697.173	4785.635	4715.825	0.887	0.432	<0.001	9.2/11.9/13.1/31.6/34.2

As described in Figure 1, class 1 (low readiness-comprehensively weak; *n* = 103, 25.0%) showed the lowest scores across personal status, adaptive ability, and expected social support. Class 2 (moderate readiness-support advantage; *n* = 141, 34.2%) showed moderate personal status and adaptive ability with high expected social support. Class 3 (high readiness-adaptation leading; *n* = 168, 40.8%) showed the highest personal status and adaptive ability, with comparatively lower expected social support than Class 2.

**Figure 1:**
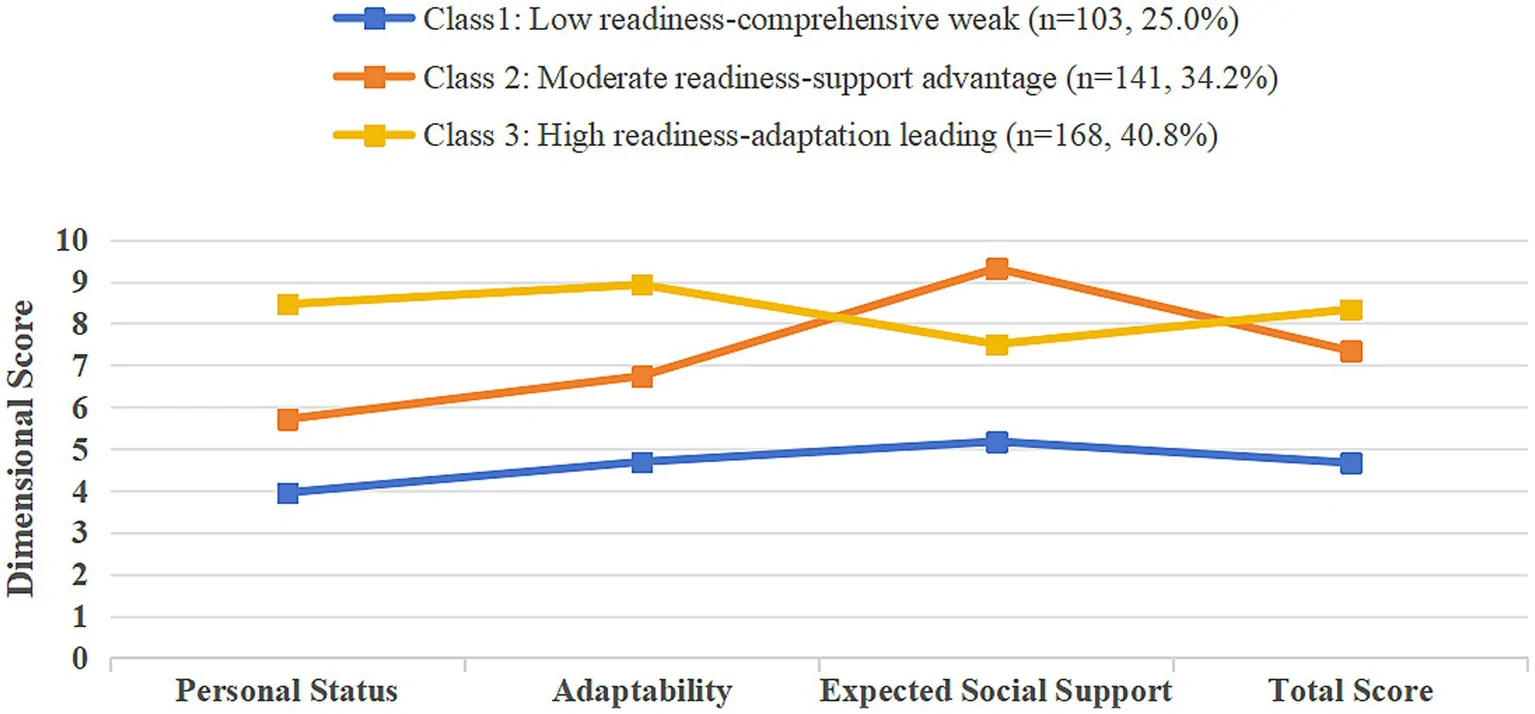
Distribution of characteristics across three latent profiles for discharge readiness in orthognathic patients (*n* = 412).

### 3.3 Univariate analysis of latent profiles of readiness for hospital discharge in orthognathic surgery patients

Univariate analyses revealed significant differences among the three latent profiles with respect to quality of discharge teaching scores, orthognathic quality-of-life scores, living status, household income level, educational attainment, and surgical type (all *p* < 0.05). Post-hoc pairwise comparisons indicated that the low readiness-comprehensively weak profile differed from the other profiles on QDTS and OQLQ scores after multiplicity adjustment; the moderate readiness-support advantage and high readiness-adaptation leading profiles also differed on QDTS and OQLQ scores. These comparisons were treated as descriptive and were not used as a substitute for multivariable modeling. Detailed results are presented in Table 3.

**Table 3:** General information and univariate analysis of latent profiles of discharge readiness among orthognathic patients (*n* = 412).

Variables	Total (*n* = 412)	Class 1 (*n* = 103)	Class 2 (*n* = 141)	Class 3 (*n* = 168)	χ^2^/H	*p*	Adjusted pairwise comparisons
Type of Surgery					χ^2^ = 60.677	<0.001	C1-C2 < 0.001; C1-C3 < 0.001; C2-C3 < 0.001
Bimaxillary surgery	237 (57.5)	48 (46.6)	98 (69.5)	91 (54.2)			
Bimaxillary surgery & genioplasty	94 (22.8)	46 (44.7)	26 (18.4)	22 (13.1)			
Monomaxillary and others	81 (19.7)	9 (8.7)	17 (12.1)	55 (32.7)			
Residence status					χ^2^ = 16.110	<0.001	C1-C2 < 0.001; C1-C3 = 0.151; C2-C3 = 0.008
Living alone	114 (27.7)	40 (38.8)	23 (16.3)	51 (30.4)			
Not living alone	298 (72.3)	63 (61.2)	118 (83.7)	117 (69.6)			
Monthly household income					χ^2^ = 17.489	0.002	C1-C2 = 0.032; C1-C3 = 0.001; C2-C3 = 0.296
≤5,000	85 (20.6)	32 (31.1)	25 (17.7)	28 (16.7)			
5,000–10,000	150 (36.4)	42 (40.8)	55 (39.0)	53 (31.5)			
>10,000	177 (43.0)	29 (28.2)	61 (43.3)	87 (51.8)			
Educational attainment					χ^2^ = 24.756	<0.001	C1-C2 = 0.024; C1-C3 < 0.001; C2-C3 = 0.024
High school and below	77 (18.7)	23 (22.3)	33 (23.4)	21 (12.5)			
Undergraduate	241 (58.5)	71 (68.9)	78 (55.3)	92 (54.8)			
Postgraduate and above	94 (22.8)	9 (8.7)	30 (21.3)	55 (32.7)			
Total Score for QDTS	140.00 (125.00, 152.00)	119.00 (106.00, 126.00)	149.00 (141.00, 157.00)	143.00 (131.00, 153.00)	H = 176.167	<0.001	C1-C2 < 0.001; C1-C3 < 0.001; C2-C3 < 0.001
Total Score for OQLQ	51.00 (45.00, 59.00)	59.00 (50.00, 66.00)	50.00 (46.00, 58.00)	48.00 (43.00, 54.00)	H = 50.862	<0.001	C1-C2 < 0.001; C1-C3 < 0.001; C2-C3 = 0.004

Full univariate analysis including non-significant variables is provided in Supplementary Table S1. Numbers outside parentheses are counts, numbers inside parentheses are percentages (%). Categorical variables were compared using chi-square tests, and ordinal/scale variables were compared using the Kruskal–Wallis test. These subgroup comparisons were conducted to describe between-profile heterogeneity and to assess the external validity of the latent profile solution. Pairwise comparisons were performed only for variables with significant omnibus tests. Dunn-Holm tests were used for continuous variables after Kruskal-Wallis tests, and multiplicity-adjusted pairwise comparisons were used for categorical variables. C1 = low readiness-comprehensively weak; C2 = moderate readiness-support advantage; C3 = high readiness-adaptation leading.

### 3.4 Multivariate logistic regression analysis of readiness for hospital discharge in orthognathic surgery patients

Class 1 (low readiness-comprehensively weak) was used as the reference category in the primary multinomial logistic regression model. QDTS and OQLQ total scores were entered as continuous variables per 10-point increase; residence status, household income, educational attainment, and surgery type were entered using indicator coding with the reference categories shown in Table 4. The global likelihood-ratio test was significant (chi-square = 360.578, df = 18, *p* = <0.001), and the Nagelkerke pseudo-R-squared was 0.659. The maximum variance inflation factor was 1.83, suggesting no serious multicollinearity. The results of the multivariate logistic regression analysis are shown in Table 5.

**Table 4:** Multivariate logistic regression analysis assignment table.

Variables	Assignment
Residence status	Living alone = reference; not living alone = 1
Household income level	≤5,000 CNY/month = reference; 5,000–10,000 and >10,000 CNY/month entered as indicator variables
Educational attainment	High school and below = reference; undergraduate and postgraduate or above entered as indicator variables
Type of surgery	Monomaxillary surgery and monomaxillary surgery with genioplasty = reference; bimaxillary surgery and bimaxillary surgery with genioplasty entered as indicator variables
QDTS total score	Continuous variable, rescaled per 10-point increase
OQLQ total score	Continuous variable, rescaled per 10-point increase; higher scores indicate poorer quality of life

**Table 5:** Multivariate analysis of latent profiles of discharge readiness among orthognathic patients (*n* = 412).

Variables	B	SE	*p*	OR	95% CI
Class 2 moderate readiness-support advantage vs. Class 1					
Constant	−18.247	2.587	<0.001	-	-
QDTS total score, per 10-point increase	1.730	0.199	<0.001	5.639	3.815–8.335
OQLQ total score, per 10-point increase	−0.990	0.245	<0.001	0.372	0.230–0.601
Not living alone	0.961	0.460	0.037	2.615	1.062–6.439
Income 5,000–10,000 CNY/month	0.454	0.559	0.417	1.575	0.526–4.712
Income >10,000 CNY/month	1.593	0.582	0.006	4.917	1.572–15.376
Undergraduate	0.278	0.508	0.584	1.321	0.488–3.577
Postgraduate and above	0.769	0.691	0.266	2.157	0.557–8.359
Bimaxillary surgery	−0.100	0.662	0.880	0.905	0.247–3.310
Bimaxillary surgery & genioplasty	−1.559	0.721	0.031	0.210	0.051–0.865
Class 3 high readiness-adaptation leading vs. Class 1					
Constant	−9.452	2.330	<0.001	-	-
QDTS total score, per 10-point increase	1.403	0.189	<0.001	4.067	2.805–5.896
OQLQ total score, per 10-point increase	−1.520	0.248	<0.001	0.219	0.134–0.356
Not living alone	0.181	0.433	0.676	1.198	0.513–2.800
Income 5,000–10,000 CNY/month	0.012	0.539	0.983	1.012	0.352–2.912
Income >10,000 CNY/month	1.680	0.553	0.002	5.368	1.817–15.861
Undergraduate	0.818	0.506	0.106	2.266	0.841–6.109
Postgraduate and above	1.960	0.677	0.004	7.099	1.884–26.750
Bimaxillary surgery	−1.476	0.619	0.017	0.229	0.068–0.770
Bimaxillary surgery & genioplasty	−3.031	0.685	<0.001	0.048	0.013–0.185

Class 1 = low readiness-comprehensively weak; Class 2 = moderate readiness-support advantage; Class 3 = high readiness-adaptation leading. Class 1 was the reference category. ORs for QDTS and OQLQ represent a 10-point increase. Income, educational attainment, and surgery type were modeled as indicator variables.

Sensitivity analyses accounting for classification uncertainty confirmed the robustness of our primary findings (Supplementary Table S1). The high-certainty analysis, which retained 386 participants (93.7%) with a maximum posterior probability $\ge$ 0.80, and the posterior-probability-weighted model both yielded effect sizes and significance levels for QDTS, OQLQ, higher socioeconomic status, and surgical extent that closely mirrored the primary model. However, the sensitivity models did attenuate the previously significant effect of not living alone on Class 2 membership ($P$ > 0.05 in both sensitivity models), suggesting this specific association was sensitive to classification error. Given that these robust models ultimately still rely on assigned or weighted membership probabilities, all reported estimates represent observational associations rather than causal effects.

## 4 Discussion

### 4.1 Orthognathic patients demonstrated a moderate level of readiness for hospital discharge with significant heterogeneity

Readiness for hospital discharge reflects patients’ perceived preparedness to leave the hospital and manage the transition from hospital-based care to home-based recovery. It is not limited to physical recovery at the time of discharge; rather, it integrates patients’ current physical condition, self-care knowledge, coping and adaptive ability, and anticipated support after returning home. For orthognathic surgery patients, this construct is particularly relevant because discharge often occurs before complete resolution of swelling, pain, oral functional limitation, dietary restriction, and psychological adaptation to facial changes. Therefore, a patient may appear clinically stable for discharge but still feel insufficiently prepared for self-management at home. The heterogeneity identified in this study indicates that discharge readiness should be understood as a multidimensional and profile-specific construct, rather than as a single overall score.

This study found that the overall readiness for hospital discharge among orthognathic patients was at a moderate level, which is consistent with previous findings reported Abu et al. (24). The median RHDS score was 7.50 (5.98, 8.25), and latent profile analysis further identified three distinct types of readiness profiles. Previous studies have shown that although orthognathic surgery significantly improves oral health-related quality of life and psychosocial well-being in the long term, the immediate postoperative period remains a critical stage requiring substantial physical and psychological adaptation (1, 25). Therefore, adequate preparation for discharge is particularly important for orthognathic surgery patients. Importantly, the present findings highlight that orthognathic patients cannot be regarded as a homogeneous population with respect to discharge preparedness. Considerable variability exists in clinical characteristics, psychosocial resources, educational attainment, socioeconomic status, and access to discharge education. Such differences may influence patients’ perceptions of recovery readiness and their capacity to manage postoperative rehabilitation. Consistent with person-centered research employing latent profile analysis in chronic disease management, psychological adaptation, and nursing science, the current findings support the existence of clinically meaningful subgroups that may require differentiated nursing strategies and tailored transitional care interventions (26, 27).

### 4.2 Latent profile analysis of readiness for hospital discharge of orthognathic patients

The present study identified three distinct latent profiles of discharge readiness among orthognathic patients: the low readiness-comprehensively weak type, the moderate readiness-support advantage type, and the high readiness-adaptation leading type. These profiles exhibited markedly different patterns across personal status, adaptive ability, and expected social support, indicating that discharge readiness is not merely a matter of degree but also reflects qualitatively different configurations of recovery resources and coping capacities. The identification of these profiles provides a more nuanced understanding of discharge readiness and offers a foundation for profile-based precision nursing interventions.

#### 4.2.1 Low readiness-comprehensively weak type

Approximately one-quarter of participants were classified into the low readiness-comprehensively weak type. Patients in this subgroup exhibited consistently low scores across all dimensions of the RHDS, indicating deficits in physical recovery, self-management confidence, adaptive capacity, and perceived support resources. Consequently, these patients may be particularly vulnerable to adverse post-discharge outcomes. Several factors may contribute to the characteristics of this profile. Orthognathic surgery frequently results in temporary impairments in eating, speaking, oral function, and daily activities, thereby increasing dependence on caregivers and reducing confidence in independent recovery. Furthermore, uncertainty regarding postoperative appearance, rehabilitation progress, and potential complications may heighten psychological distress and diminish perceptions of readiness (28, 29). Previous studies have demonstrated that inadequate discharge readiness is associated with poorer self-care behaviors, reduced treatment adherence, heightened anxiety, and increased healthcare utilization following discharge (30). From a clinical perspective, this subgroup represents a priority target for intervention. Conventional discharge education may be insufficient to address their complex needs. Instead, comprehensive transitional care approaches incorporating multidisciplinary discharge planning, individualized rehabilitation guidance, psychological assessment, telehealth support, and continuous follow-up should be implemented to facilitate recovery and reduce post-discharge risks.

#### 4.2.2 Moderate readiness-support advantage type

Approximately one-third of participants were categorized as the moderate readiness-support advantage type. These patients reported relatively high levels of expected social support but only moderate levels of personal status and adaptive ability, suggesting a reliance on external resources during recovery. This finding may reflect the sociocultural characteristics of the study population. Most participants were young adults, many of whom resided with family members. Within the Chinese cultural context, family involvement often plays a central role in postoperative care by providing emotional encouragement, practical assistance, and health-related guidance, thereby enhancing patients’ perceived readiness for discharge (31). However, the present findings also indicate that social support alone may be insufficient to achieve optimal discharge readiness. Excessive dependence on caregivers may inadvertently limit opportunities for developing self-management competence and independent coping skills. Therefore, nursing interventions for this subgroup should extend beyond strengthening support networks and focus on enhancing self-efficacy, promoting active participation in rehabilitation, and fostering autonomous disease-management behaviors.

#### 4.2.3 High readiness-adaptation leading type

The high readiness-adaptation leading profile represented the largest subgroup. Patients within this profile demonstrated the highest levels of personal status and adaptive ability, despite not reporting the highest expected social support. This pattern suggests a possible role of internal adaptive resources in discharge readiness, but the present cross-sectional data cannot establish whether these resources are more influential than external support. Future studies should directly test the relative contribution of internal and external resources and examine whether interventions that strengthen adaptive capacity improve post-discharge outcomes.

A recent study by Xu et al., (32) also applied latent profile analysis to discharge readiness and identified three profiles among older patients with hip fractures: low readiness-overall weakness, medium readiness-support advantage, and high readiness-adaptation leadership. The similarity between these profiles and the present findings suggests that discharge readiness may show a reproducible pattern across different surgical populations: one subgroup with broadly insufficient readiness, one subgroup whose perceived support is relatively stronger than personal or adaptive readiness, and one subgroup with relatively stronger self-perceived recovery and adaptive capacity. However, the present study extends this prior work to orthognathic surgery patients, a younger and clinically distinct population facing specific challenges such as postoperative facial swelling, oral function limitation, dietary modification, speech difficulty, appearance-related concerns, and prolonged orthodontic-surgical treatment adaptation. Therefore, although the profile labels were informed by the relative RHDS dimension patterns and are conceptually consistent with previous LPA findings, they should be interpreted in the specific clinical context of orthognathic surgery rather than as direct replication of profiles from older orthopedic patients.

### 4.3 Association between quality of discharge teaching and readiness-for-discharge profiles

Quality of discharge teaching was strongly associated with assigned readiness-for-discharge profile membership. Because QDTS, OQLQ, and RHDS were measured concurrently and by self-report, the temporal order of these associations cannot be established. Therefore, discharge teaching quality should be interpreted as a modifiable clinical factor associated with readiness profiles rather than as a demonstrated causal determinant. Patients in the low readiness-comprehensively weak profile reported lower QDTS scores, suggesting that both the amount of discharge information actually received and the perceived effectiveness of teaching may be important targets for future discharge-preparation programs. These implications are hypothesis-generating and should be tested in longitudinal or interventional studies.

### 4.4 Association between orthognathic-related quality of life and readiness for hospital discharge profile

The present study identified orthognathic-related quality of life as an important factor associated with discharge readiness profile membership. Patients reporting poorer quality of life were more likely to be classified into the low readiness-comprehensively weak type, whereas those with better quality of life were more frequently assigned to the high readiness-adaptation leading type. These findings suggest that discharge readiness is influenced not only by objective physical recovery but also by patients’ subjective perceptions of their functional status, psychosocial well-being, and overall adaptation to the postoperative recovery process. The observed association is consistent with previous evidence linking quality of life to postoperative adaptation among orthognathic patients. Duarte et al., (1) reported that improvements in oral health-related quality of life following orthognathic surgery were accompanied by enhanced social functioning and psychological well-being. Similarly, Ebenezer et al., (25) found that postoperative satisfaction and quality-of-life improvements were strongly associated with successful adaptation to treatment outcomes. Collectively, these findings support the notion that quality of life represents an important indicator of patients’ capacity to cope with recovery-related challenges and achieve successful postoperative adjustment. Several potential mechanisms may explain this relationship. First, poorer quality of life may reflect greater postoperative symptom burden, these symptoms can directly undermine patients’ perceptions of physical readiness for discharge. Second, quality of life is closely associated with psychological adaptation. Patients who continue to experience concerns regarding facial appearance, social acceptance, or recovery uncertainty may exhibit heightened anxiety and reduced confidence in their ability to cope with home-based rehabilitation. Such psychological distress may negatively influence adaptive ability. Third, quality of life may indirectly influence discharge readiness through self-efficacy. Interestingly, the OQLQ dimensions most closely related to discharge readiness in the present study appeared to be oral function and social functioning. This observation may reflect the unique characteristics of orthognathic recovery. Difficulties in chewing, speaking, and social interaction are often highly visible and directly affect daily living experiences (33), making them particularly influential in shaping perceptions of recovery preparedness. Therefore, postoperative nursing interventions should extend beyond symptom management and include psychosocial support, body image counseling, and strategies aimed at promoting social reintegration (34).

### 4.5 Association of socioeconomic resources and surgical complexity with potential categories of readiness for hospital discharge

Surgery type was associated with assigned profile membership. In the regression model, bimaxillary surgery with genioplasty was less likely to be represented in higher-readiness profiles than the reference category of monomaxillary surgery and monomaxillary surgery with genioplasty. This association is clinically plausible because more extensive procedures may be accompanied by greater tissue trauma, swelling, functional limitation, and uncertainty during early recovery. However, surgery type was categorized for analysis, and the findings should be interpreted cautiously until validated in larger multicenter samples.

### 4.6 Implications for profile-based precision discharge nursing interventions

One of the most important contributions of this study lies in its identification of distinct discharge readiness profiles that may serve as the foundation for precision nursing interventions.

The concept of precision nursing has gained increasing attention in recent years and emphasizes the delivery of individualized interventions based on patients’ unique characteristics, risk profiles, and healthcare needs. Consistent with recent latent profile studies conducted in chronic disease management, nursing competence development, and violence prevention training, person-centered classification approaches may facilitate more efficient allocation of healthcare resources and improve intervention effectiveness (26, 30). By identifying patients with distinct discharge readiness patterns, nurses can tailor interventions according to specific recovery barriers and support requirements.

For patients classified in the low readiness-comprehensively weak type, enhanced transitional-care interventions should be prioritized. These patients constitute a high-risk subgroup characterized by relatively poor physical status, limited adaptive capacity, and inadequate perceived support resources. Because orthognathic surgery patients commonly experience postoperative facial swelling, dietary restrictions, speech difficulties, and prolonged functional recovery, insufficient discharge readiness may increase the likelihood of self-management difficulties after discharge. Accordingly, discharge preparation should begin early during hospitalization and include individualized discharge counseling, repeated skills training, structured rehabilitation planning, psychological assessment, family caregiver engagement, and scheduled follow-up after discharge (35). Given their lower adaptive ability and weaker support systems, continuity of care is particularly important. Technology-assisted nursing strategies, including mobile health applications, teleconsultation services, video-based educational resources, and remote symptom monitoring, may provide additional support and facilitate early identification of recovery-related problems (36, 37).

For patients in the moderate readiness-support advantage type, nursing interventions should focus on enhancing self-management competence and rehabilitation engagement. Although these patients reported relatively strong social support, their adaptive ability remained comparatively limited. This finding suggests that external support alone may be insufficient to ensure successful recovery if patients lack confidence and practical skills for independent self-management. Therefore, discharge education should extend beyond information provision and emphasize self-efficacy enhancement, problem-solving skills, symptom-management strategies, and behavioral activation (38). Family-centered nursing interventions may be particularly beneficial for this subgroup, as caregivers can facilitate the gradual transition from dependence on external support to autonomous rehabilitation management (39).

Patients classified in the High readiness-Adaptation Leading type demonstrated relatively strong adaptive capacity and recovery readiness. For this subgroup, nursing care may shift from intensive supervision toward empowerment-based management and self-management optimization. Nurses can encourage active participation in recovery planning, self-monitoring of rehabilitation progress, and engagement in patient-support networks (40). In addition, these patients may represent potential peer-support resources for other orthognathic patients undergoing similar treatment experiences. Previous evidence suggests that peer-support interventions may enhance self-confidence, reduce uncertainty, and improve treatment adherence during long-term rehabilitation processes (41).

Overall, the findings suggest that profile-informed discharge assessment may help nurses recognize different patterns of discharge readiness and identify patients who may need additional preparation before returning home. The proposed nursing strategies should be viewed as clinical implications and hypotheses for future testing rather than as proven effects of the present cross-sectional study.

### 4.7 Strengths and limitations

This study contributes to the growing body of evidence on transitional care in surgical populations. To the best of our knowledge, this is one of the first studies to examine readiness for hospital discharge in orthognathic patients. By applying latent profile analysis, this study moved beyond conventional variable-centered approaches and identified substantial heterogeneity in discharge readiness among orthognathic patients. These findings provide practical evidence to support individualized discharge planning strategies and profile-based transitional care pathways within ERAS frameworks.

Several limitations of this study should be acknowledged. First, the cross-sectional design precludes causal inference, and QDTS, OQLQ, and RHDS were measured concurrently by self-report, which may introduce temporal ambiguity and common-method bias. Second, this was a single-center study using convenience sampling, and the sample was predominantly young, female, urban, highly educated, and self-paying; patients aged 50 years or older and those with education below junior-high level were excluded. These features restrict the generalizability of the findings. Third, electronic questionnaire administration may have introduced selection effects, and responses completed independently may differ from investigator-assisted responses. Fourth, although the three-profile solution showed good separation, the solution has not been externally validated in an independent sample. Fifth, subsequent regression analyses used assigned most-likely profile membership; classification uncertainty was addressed through sensitivity analyses but cannot be completely eliminated. Finally, this study did not assess post-discharge outcomes, so profile-specific nursing strategies should be tested in future longitudinal and interventional studies.

## 5 Conclusion

This study found that readiness for hospital discharge among patients undergoing orthognathic surgery was moderate and heterogeneous. Three assigned profiles were identified: low readiness-comprehensively weak, moderate readiness-support advantage, and high readiness-adaptation leading. Quality of discharge teaching, orthognathic-related quality of life, living status, household income, educational attainment, and surgery type were associated with assigned profile membership. These findings suggest that future discharge-preparation programs may consider profile-informed assessment and support, particularly for patients with lower readiness, poorer quality of life, fewer socioeconomic resources, or more extensive surgical procedures. Longitudinal and intervention studies are needed to determine whether such strategies improve post-discharge recovery and related outcomes.

## Data Availability

The original contributions presented in the study are included in the article/supplementary material, further inquiries can be directed to the corresponding author.
