# Supplementary material for: Latent profiles and associated factors of readiness for hospital discharge among patients undergoing orthognathic surgery: a cross-sectional study
Source: Front Public Health. 2026 Sep 24;14:1925469. doi: 10.3389/fpubh.2026.1925469 (PMC13648036; doi:10.3389/fpubh.2026.1925469)
Supplement: Supplementary file 1 [file Supplementary_file_1.pdf]

Supplementary File: Mplus and SPSS Syntax for Statistical Analyses

1. Mplus Syntax for Latent Profile Analysis

The latent profile analysis (LPA) was conducted using the three nonredundant RHDS dimension mean scores as continuous indicators: personal status, adaptive ability, and expected social support. The overall RHDS mean score was not entered as an LPA indicator.

The following syntax shows the three-profile model retained in the final manuscript. Models with one to five profiles were estimated by changing the number in the CLASSES command.

```
TITLE:
  Latent profile analysis of readiness for hospital discharge
  among patients undergoing orthognathic surgery.

DATA:
  FILE = rhds_lpa_data.dat;

VARIABLE:
  NAMES =
    id
    personal_status
    adaptive_ability
    expected_support;

  USEVARIABLES =
    personal_status
    adaptive_ability
    expected_support;

  CLASSES = c(3);

  MISSING = ALL (-999);

ANALYSIS:
  TYPE = MIXTURE;
  ESTIMATOR = MLR;
  STARTS = 1000 250;
  STITERATIONS = 20;
  LRTSTARTS = 0 0 500 100;
  PROCESSORS = 4;

MODEL:
  %OVERALL%
    personal_status adaptive_ability expected_support;
    personal_status WITH adaptive_ability@0;
    personal_status WITH expected_support@0;
    adaptive_ability WITH expected_support@0;
```

```
   %c#1%
      [personal_status adaptive_ability expected_support];

   %c#2%
      [personal_status adaptive_ability expected_support];

   %c#3%
      [personal_status adaptive_ability expected_support];

OUTPUT:
   TECH1 TECH4 TECH11 TECH14;
   SVALUES;
   STANDARDIZED;

SAVEDATA:
   FILE = lpa_3class_prob.dat;
   SAVE = CPROBABILITIES;
```

For model enumeration, the following line was changed successively:

```
CLASSES = c(1);
CLASSES = c(2);
CLASSES = c(3);
CLASSES = c(4);
CLASSES = c(5);
```

For the four-profile model, additional random starts were used to examine the stability of the best log-likelihood:

```
ANALYSIS:
   TYPE = MIXTURE;
   ESTIMATOR = MLR;
   STARTS = 2000 500;
   STITERATIONS = 20;
   LRTSTARTS = 0 0 500 100;
   PROCESSORS = 4;
```

2. SPSS Syntax for Descriptive Statistics and Group Comparisons

```
GET FILE='analysis_dataset.sav'.

DATASET NAME main.

* Profile variable:
* 1 = low readiness-comprehensively weak.
* 2 = moderate readiness-support advantage.
* 3 = high readiness-adaptation leading.
```

```
VALUE LABELS profile
  1 'Low readiness-comprehensively weak'
  2 'Moderate readiness-support advantage'
  3 'High readiness-adaptation leading'.

FREQUENCIES VARIABLES=
  gender age_group residence_area marital living_status
  education income3 insurance occupation surgery3 profile
  /ORDER=ANALYSIS.

EXAMINE VARIABLES=
  rhds_mean personal_status adaptive_ability expected_support
  qdts_total qdts_need qdts_actual qdts_skill
  oqlq_total oqlq_oral oqlq_aesthetic oqlq_deformity oqlq_social
  BY profile
  /STATISTICS DESCRIPTIVES
  /PLOT NONE
  /PERCENTILES(25,50,75)
  /MISSING LISTWISE.
```

3. SPSS Syntax for Kruskal-Wallis Tests and Pairwise Comparisons

```
NPTESTS
  /INDEPENDENT TEST
    (qdts_total oqlq_total
     qdts_need qdts_actual qdts_skill
     oqlq_oral oqlq_aesthetic oqlq_deformity oqlq_social)
    GROUP(profile)
    KRUSKAL_WALLIS(COMPARE=PAIRWISE)
  /MISSING SCOPE=ANALYSIS USERMISSING=EXCLUDE
  /CRITERIA ALPHA=0.05.
```

Pairwise comparisons were interpreted with multiplicity adjustment. These results were reported descriptively and were not used as a substitute for the multivariable multinomial logistic regression model.

4. SPSS Syntax for Categorical Group Comparisons

```
CROSSTABS
  /TABLES=
    gender age_group residence_area marital living_status
    education income3 insurance occupation surgery3
    BY profile
  /STATISTICS=CHISQ
  /CELLS=COUNT COLUMN EXPECTED
  /COUNT ROUND CELL.
```

For variables with small expected cell counts, Fisher exact or Monte Carlo exact tests were

requested as appropriate:

```
CROSSTABS
   /TABLES=surgery3 BY profile
   /STATISTICS=CHISQ
   /CELLS=COUNT COLUMN EXPECTED
   /COUNT ROUND CELL
   /METHOD=MONTECARLO CIN(99) SAMPLES(10000).
```

5. SPSS Syntax for Multinomial Logistic Regression

Class 1, the low readiness-comprehensively weak profile, was used as the reference outcome category. QDTS and OQLQ total scores were rescaled per 10-point increase to improve clinical interpretability.

```
COMPUTE qdts10 = qdts_total / 10.
COMPUTE oqlq10 = oqlq_total / 10.
EXECUTE.

VALUE LABELS profile
   1 'Low readiness-comprehensively weak'
   2 'Moderate readiness-support advantage'
   3 'High readiness-adaptation leading'.

NOMREG profile (BASE=FIRST ORDER=ASCENDING)
   WITH qdts10 oqlq10
   /CRITERIA CIN(95) DELTA(0) MXITER(100) MXSTEP(5)
      LCONVERGE(0) PCONVERGE(0.000001) SINGULAR(0.00000001)
   /MODEL qdts10 oqlq10 living_status income3 education surgery3
   /INTERCEPT INCLUDE
   /PRINT PARAMETER SUMMARY LRT CPS STEP MFI.
```

Indicator coding was used for categorical predictors, with the following reference categories:

Residence status: living alone

Monthly household income: <=5000 CNY/month

Educational attainment: high school and below

Surgery type: monomaxillary surgery and others

6. SPSS Syntax for Multicollinearity Assessment

Multicollinearity among predictors was assessed using variance inflation factors from an auxiliary linear regression model.

```
REGRESSION
   /DEPENDENT qdts10
   /METHOD=ENTER oqlq10 living_status income3 education surgery3
   /STATISTICS COEFF OUTS R ANOVA COLLIN TOL.
```

The maximum variance inflation factor was used to evaluate multicollinearity.

7. Sensitivity Analysis 1: High-Certainty Classification Subsample

Participants with maximum posterior probability ≥0.80 were retained for the high-certainty sensitivity analysis.

```
USE ALL.
COMPUTE high_certainty = (max_postprob >= 0.80).
EXECUTE.

FILTER BY high_certainty.

NOMREG profile (BASE=FIRST ORDER=ASCENDING)
  WITH qdts10 oqlq10
  /CRITERIA CIN(95) DELTA(0) MXITER(100) MXSTEP(5)
    LCONVERGE(0) PCONVERGE(0.000001) SINGULAR(0.00000001)
  /MODEL qdts10 oqlq10 living_status income3 education surgery3
  /INTERCEPT INCLUDE
  /PRINT PARAMETER SUMMARY LRT CPS STEP MFI.

FILTER OFF.
USE ALL.
EXECUTE.
```

8. Sensitivity Analysis 2: Posterior-Probability-Weighted Model

Posterior probabilities exported from Mplus were used to construct weighted sensitivity models. Separate weighted multinomial models were estimated using posterior class-membership probabilities to evaluate whether the main findings were robust to classification uncertainty.

```
* Example: posterior probabilities exported from Mplus.
* post_low      = posterior probability for Class 1.
* post_moderate = posterior probability for Class 2.
* post_high     = posterior probability for Class 3.

WEIGHT BY post_low.
NOMREG profile (BASE=FIRST ORDER=ASCENDING)
  WITH qdts10 oqlq10
  /MODEL qdts10 oqlq10 living_status income3 education surgery3
  /INTERCEPT INCLUDE
  /PRINT PARAMETER SUMMARY LRT CPS STEP MFI.

WEIGHT OFF.

WEIGHT BY post_moderate.
NOMREG profile (BASE=FIRST ORDER=ASCENDING)
  WITH qdts10 oqlq10
  /MODEL qdts10 oqlq10 living_status income3 education surgery3
  /INTERCEPT INCLUDE
  /PRINT PARAMETER SUMMARY LRT CPS STEP MFI.
```

```
WEIGHT OFF.

WEIGHT BY post_high.
NOMREG profile (BASE=FIRST ORDER=ASCENDING)
  WITH qdts10 oqlq10
  /MODEL qdts10 oqlq10 living_status income3 education surgery3
  /INTERCEPT INCLUDE
  /PRINT PARAMETER SUMMARY LRT CPS STEP MFI.

WEIGHT OFF.
```

Because posterior-probability weighting is a sensitivity analysis rather than a fully bias-adjusted three-step model, the results were interpreted as robustness checks rather than definitive causal estimates.

9. Notes on Interpretation

The primary regression treated most-likely assigned profile membership as the outcome. Therefore, all regression results were interpreted as associations with assigned profile membership, not as causal effects. Sensitivity analyses were conducted to evaluate whether the main results were robust to classification uncertainty.
